# Impact of a real-time electronic persistent hyperglycemia alert on glucose control in the ICU

**DOI:** 10.1186/cc12393

**Published:** 2013-03-19

**Authors:** K Colpaert, S Oeyen, T van den Driessche, C Danneels, J Decruyenaere

**Affiliations:** 1 Ghent University Hospital, Ghent, Belgium

## Introduction

Hyperglycemia is frequently encountered in critically ill patients, and associated with adverse outcome. Improvement of glucose protocol adherence may be accomplished using electronic alerts. We configured a non-intrusive real-time electronic alert, called a GLYC sniffer, as part of our Intensive Care Information System (ICIS) that continuously evaluates the occurrence of persistent hyperglycemia and hypoglycemia. The GLYC sniffer is configured in such a way to give an alert: if two consecutive glucose values are >150 mg/dl, with a minimum interval of 60 minutes; or if a glucose value is <80 mg/dl. We wanted to evaluate whether the GLYC sniffer would improve the glucose control.

## Methods

A single-center, prospective intervention study during a 6-month period in our 22-bed surgical ICU. Two study phases were compared: a 3-month pre-alert phase with no alerting to the nurses, and a 3-month intervention phase where the GLYC sniffer was alerting through the Clinical Notification System of our ICIS.

## Results

A total of 652 different patients having a total of 699 admission episodes was recorded during the study period. There were no significant differences between the two study groups regarding baseline demographic data, first glucose value upon admission. A total of 2,335 GLYC sniffer alerts were recorded during the whole study period: 84.3% persistent hyperglycemia alerts (1,021 in the pre-alert group, 948 in the alert group), and 15.7% hypoglycemia alerts (139 vs. 227). A significantly lower percentage of glucose values in the alert group were hyperglycemic (19.5% vs. 26.5%, *P *0.001). The proportion of persistent hyperglycemic values (i.e. consecutive glucose values exceeding the limit of 150 mg/dl) was significantly lower in the alert group (9.9% vs. 15.4%, *P *0.001). The patients in the alert group spend significantly more time within the set target glucose interval of 80 to 150 mg/dl (82.3% vs. 75.0%, *P *= 0.009). A significantly lower proportion of patients experienced a new-onset hypoglycemic event (<80 mg/dl) in the alert group (19.3% vs. 26.2%, *P *= 0.04).

## Conclusion

A real-time electronic persistent glycemia sniffer resulted in a significantly higher proportion of normoglycemia, without increasing the variability. Furthermore, hypoglycemic events occurred less frequently, and were resolved more timely. Smart alerting is able to improve quality of care, while diminishing the problem of alert fatigue.

